# Moving Object Localization Based on UHF RFID Phase and Laser Clustering

**DOI:** 10.3390/s18030825

**Published:** 2018-03-09

**Authors:** Yulu Fu, Changlong Wang, Ran Liu, Gaoli Liang, Hua Zhang, Shafiq Ur Rehman

**Affiliations:** 1 School of Information Engineering, Southwest University of Science and Technology, Mianyang 621010, China; fyl4499@163.com (Y.F.); young.white@163.com (C.W.); m18381669557@163.com (G.L.); huazhang@swust.edu.cn (H.Z.); shafiq@luawms.edu.pk (S.U.R); 2 Engineering Product Development, Singapore University of Technology and Design, Singapore 487372, Singapore; 3 Department of Computer Science, Lasbela University of Agriculture, Water and Marine Sciences, Balochistan 90150, Pakistan

**Keywords:** RFID, phase difference, laser clustering, velocity matching, particle filter

## Abstract

RFID (Radio Frequency Identification) offers a way to identify objects without any contact. However, positioning accuracy is limited since RFID neither provides distance nor bearing information about the tag. This paper proposes a new and innovative approach for the localization of moving object using a particle filter by incorporating RFID phase and laser-based clustering from 2d laser range data. First of all, we calculate phase-based velocity of the moving object based on RFID phase difference. Meanwhile, we separate laser range data into different clusters, and compute the distance-based velocity and moving direction of these clusters. We then compute and analyze the similarity between two velocities, and select K clusters having the best similarity score. We predict the particles according to the velocity and moving direction of laser clusters. Finally, we update the weights of the particles based on K clusters and achieve the localization of moving objects. The feasibility of this approach is validated on a Scitos G5 service robot and the results prove that we have successfully achieved a localization accuracy up to 0.25 m.

## 1. Introduction

Recent documents show a growing interest in indoor localization due to the high demand of location-based services (LBS) [1,2], for example asset tracking and indoor guidance. Extensive research have been done regarding vision-based and range-based sensors. Vision-based localization is challenging as it needs the object to be in the line of sight and also requires complex algorithms to recognize the object. The advantages of RFID (Radio Frequency Identification) provide a way to overcome these difficulties, as RFID has some unique qualities like an RFID tag has a unique identification code (ID), which does not require any complex recognition algorithm [3]. Secondly, compared to vision or range-based sensors, RFID can solve the occlusion issue of the object as the communication of the RFID does not to be in the line of sight. These characteristics make it convenient to use RFID for positioning of an object. In addition, RFID positioning also has the advantages of small size and fast position deduction, as compared to Bluetooth or Wi-Fi positioning techniques [4]. Therefore, RFID positioning technology is widely used in warehousing, library, supermarket, logistics assembly, and other places [5,6]. For example, Shaari et al. proposed an intelligent storage system based on RFID that uses a database to store the position and orientation of an object [7]. Lu et al. designed an RFID-based positioning system for an AGV (Automated Guided Vehicle) in a smart factory [8]. Fortinsimard et al. implemented a smart identification system based on passive RFID technology for smart homes [9]. Nur et al. designed a system that provides real-time information about the product by projecting RFID and shelf information onto a panoramic view [10]. Wang et al. proposed an RFID-based positioning approach for connected vehicles [11].

RFID systems also have some limitations like RFID can not directly obtain the distance or bearing of the tag, so one has to rely on other information to achieve the positioning task. Traditional ways of localization by RFID are commonly based on received signal strength (RSS) with the limitation of multi-path propagation issues in indoor environment, which could lead to poor localization accuracy and demands excessive training to build the model [12]. Hähnel et al. used a mobile robot to measure the signal strength of tags at different positions and constructed a probabilistic sensor model of RFID antenna to locate passive RFID tags; however, this method needs to calibrate the RFID sensor model by installing RFID tags in a well-known environment, and it is obviously time consuming [13]. Although the method used in LANDMARC gives comparatively high accuracy, it requires multiple antennas to detect the phase, which is not practical for mobile robots [14]. Many researchers have tried different ways to improve the localization accuracy based on RSS. For example, Xu et al. used the Bayesian approach and K-Nearest Neighbor to filter out the noise based on LANDMARC with a limitation of a large number of reference tag requirements in advance [15]. Yang et al. applied an improved particle filter based on the features of moving direction in a densely tagged RFID environment, but it requires a long time to build the system model [16]. Moreover, the signal’s time-of-arrival (TOA) and angel-of-arrival (AOA) can also be used for localization. Xu et al. used a TOA-based method to track the position of moving people [17]. TOA has an advantage of a simple positioning principle with small errors, but it requires strict time synchronization and high cost hardware. AOA is vulnerable to environmental factors and has limited applications [4,18]. In addition, the phase information provided by the RFID reader can be used to locate the object directly, without any explicit modeling of signal strength. However, there is an ambiguity problem in the phase extraction [19], which increases the positioning error. Sarkka et al. proposed a UHF RFID tracking system based on the phases from multiple spatially distributed antennas [20]. Some researchers also have tried to combine the RSS and phase for the localization of an object. For example, Ma et al. [21] proposed an approach to predict the position of a mobile object using the RSS and phase. Martinelli et al. proposed using the unscented Kalman filter based on the RSS and phase to locate the robot, but it requires a group of passive tags on the ceiling as landmarks [22].

In literature, RFID is also used to combine with other sensors (for example, laser range finders and visual cameras) to improve the positioning accuracy [23,24,25]. For example, Deyle et al. used visual and three-dimensional laser ranging information to construct images and combined signal strength of different angles by rotating the antenna to deduct the location of an object [26]. RFID technology is gradually applied to the field of construction [27]. Valero et al. used a 3D laser scanner and RFID technology for automatic construction of the 3D model of an environment. The fusion technique allows for identifying the main components of a structure, such as walls, floor, and windows [28]. However, this approach requires the modeling of both sensors. Zhou et al. used a new positioning system based on artificial landmarks for mobile robots. They used a laser-activated RFID tag (active tag) that has a bright LED as landmark for the positioning. The airborne laser sensor emits a laser beam to activate the tag when the robot is moving in the environment. At the same time, the robot detects the tag in the environment through the stereo vision and RFID reader, and calculates the relative position of the tag through the perspective geometry. They combined the information from multiple tags to determine the position and orientation of the robot [29]. However, this method requires the tag to be installed in the test environment in advance and the maintenance cost is high. Martin et al. used combined signals (WiFi and Digital Television) to reduce the error [30]. Liu et al. used a three-dimensional sensor model and a pair of RFID antennas at different heights to localize the tags in 3D [31]. Shirehjini et al. used RFID carpets and several peripherals of sensor to build a positioning system based on low-range passive RFID technology [32]. However, this approach requires installing RFID tags on the floor. Song et al. used a two-level extended Kalman filter (EKF) to fuse the data of sensors mounted in vehicles and RFID data for positioning of a vehicle in GPS-denied environment [33]. Xiong et al. presented a hybrid WSN-RFID system for tracking indoor objects [34]. Su et al. proposed an enhanced boundary condition approach to improve the localization accuracy of an RFID system by integrating the information from a GPS system [35].

Due to their long range, high accuracy, and fast response, laser range finders are widely used in obstacle avoidance, localization, and navigation in robotics [36,37,38]. An et al. proposed an approach that allows a robot to detect and avoid obstacles using a Vector Polar Histogram (VPH) [39]. Sun et al. proposed a noise reduction algorithm to improve ranging accuracy of a laser range finder [40]. With a laser range finder, one can directly obtain the distance and angle information of the obstacles [41,42]. For example, Vu et al. used a laser range finder to create a raster map of the environment that can be used to detect moving objects [43]. Dietmayer et al. used a model-based approach to identify and track objects in the environment [44]. However, laser-based sensors need to establish a model and complex recognition algorithms to identify the object, which usually requires a pre-training stage and expensive computational time [44,45].

The uniqueness of RFID tags makes it possible to recognize an object without any additional recognition stage like a laser range finder or visual camera always needs. Therefore, we propose an approach to combine UHF RFID technology and laser ranging information to localize a moving object. RFID tags have unique IDs that can be used to identify an object. The phase reported from the RFID is a periodic function of the distance and has the ambiguity in positioning of an object [46,47]. The phase difference can be used to infer the moving velocity of an object. On the other hand, the laser range finder can be used to measure distance and angle of the obstacles. However, it needs to recognize the object that we want to localize from a large number of objects in the laser view. The integration of RFID and laser information can lead to a more reliable and accurate positioning system. In particular, we achieve the position deduction of moving object by the fusion of RFID phase and laser clustering. We first estimate the phase-based velocity by phase difference of RFID signals. In the meantime, we segment 2d laser ranging data into clusters and estimate their velocities (i.e., distance-based velocities) and moving directions. Then, we calculate the similarity between the phase-based velocity and the distance-based velocities to analyze the similarity of both velocities. Finally, we predict and update the set of particles in a particle filter according to matching results and accomplish the localization of a moving object. Our approach does not need any signal strength information from RFID, nor does it need any modeling of the object from a laser range finder.

We organize the subsequent sections of this paper as follows. An overview of the system is described in Section 2. In Section 3, we present the details of the sensor fusion using a particle filter. We show the experimental results in Section 4 and conclude the paper with possible extensions in Section 5.

## 2. System Overview

**Figure 1:**
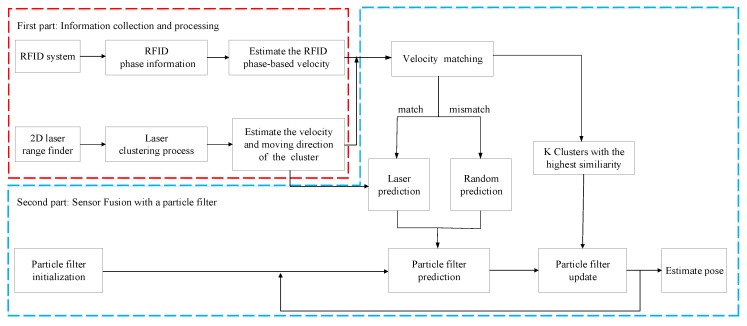
System overview.

Our approach for localization is based on the particle filter that integrates the RFID phase and laser clustering, without any modeling on signal strength. As shown in Figure 1, the whole system consists of two major parts: (1) collecting information by an RFID reader and a laser range finder on the robot; (2) fusing the RFID and laser ranging information in a particle filter for localization.

In the first part, we use the RFID reader to obtain the RFID phase of a moving object affixed with a passive tag and then calculate the phase-based velocity of the moving object by the phase difference between two successive RFID signals. It is important to note that there is a phase ambiguity during the processing of RFID phase. Therefore, we need to take measures to reduce the influence of phase ambiguity in order to estimate the phase-based velocity. After several experimental tests, we found that the phase difference of a moving object between two adjacent moments increases sharply, but the difference does not exceed $\pm 90^{\circ }$. Hence, this check helps us to reduce phase ambiguity and we can easily calculate corresponding velocity from phase difference. Meanwhile, we scan the moving object with a 2D laser range finder carried by the robot and segment the ranging data into clusters. After that, we find the neighboring clusters (two neighbors) in the continuous time and treat the two clusters as the same obstacle, which is used to estimate the distance-based velocity. This part of information acquisition and data processing is used for subsequent velocity matching and the particle filtering.

In the other part, we first calculate the similarity between phase-based velocity and the distance-based velocity and then determine the velocity matching according to a defined similarity rule. In addition, we choose the best K clusters with the best velocity similarity score to facilitate the processing of the particle filtering. To improve localization accuracy and robustness of the system, we use different prediction methods for the particle filter based on the velocity matching results: (1) If the velocity matching is successful, we can find the effective laser cluster for prediction, which is referred to as laser prediction in this paper. (2) If the velocity matching is unsuccessful, we choose another method called the random prediction method. After that, we update the particle’s weight in the particle filter using the best K clusters. Finally, we localize the moving object by iteratively performing prediction, update, and resampling of the particle filter. The role of this part is to improve the positioning efficiency and accuracy by complementing the RFID phase with laser ranging information using a particle filter.

## 3. Moving Object Localization Based on the Particle Filtering

We describe the details of the system in this section. In particular, the computation of RFID phase velocity is described in Section 3.1, the clustering of laser ranging data is presented in Section 3.2, the estimation of the velocity and moving direction of a cluster is detailed in Section 3.3, and velocity matching and the implementation using a particle filter are detailed in Section 3.4 and Section 3.5, respectively. The mathematical symbols and their meanings used in this paper are listed in Table 1.

### 3.1. Computing RFID Phase-Based Velocity

The phase obtained by RFID is a periodical function, which can be described as:

(1) $$\varphi_{t}=\frac{2\pi \cdot d_{t}}{\lambda }\cdot mod\left(2\pi \right),$$

where $\varphi_{t}$ is the signal phase at time *t*, λ is the wavelength of the receiving signal, and $d_{t}$ is the distance from RFID tag to the antenna. In practice, signal phase is influenced by the transmitter, receiver, and tag’s reflection characteristics [30], which introduce additional phase rotation. Therefore, the phase of RFID signal could be modeled as:

(2) $$\varphi_{t}=\frac{4\pi \cdot d_{t}}{\lambda }+\varphi_{T}+\varphi_{R}+\varphi_{Tag},$$

where $\varphi_{T}$ is the reader’s transmitter phase deflection, $\varphi_{r}$ is the reader’s receiver phase deflection, and $\varphi_{Tag}$ is the tag’s phase deflection.

In this paper, the moving object is affixed with a passive tag and moves at a velocity of $v_{t}^{r}$, the signal phase being received by the RFID reader continuously. We denote the phases at t−1 and *t* as $\varphi_{t-1}$ and $\varphi_{t}$, respectively; therefore, we can estimate the moving velocity of the object by the phase difference:

(3) $$v_{t}^{r}=\frac{\Delta d_{t}}{\Delta t}=\frac{d_{t}-d_{t-1}}{\Delta t}.$$

Combined with Equation (3), moving velocity of a tag can be given as:

(4) $$v_{t}^{r}=\left\{\begin{array}{cc} \frac{c}{4\pi f\Delta t}\cdot \left(\varphi_{t}-\varphi_{t-1}\right), & \left|\varphi_{t}-\varphi_{t-1}\right|<\pi ,\\ \frac{c}{4\pi f\Delta t}\cdot \left(\varphi_{t}-\varphi_{t-1}-2\pi \right), & \varphi_{t}-\varphi_{t-1}\geq \pi ,\\ \frac{c}{4\pi f\Delta t}\cdot \left(\varphi_{t}-\varphi_{t-1}+2\pi \right), & \varphi_{t}-\varphi_{t-1}\leq -\pi . \end{array}\right.$$

In this way, the influence of phase ambiguity is effectively reduced, where *c* is the velocity of light, *f* is signal frequency, and $\Delta d_{t}$ is the moving distance of the tag at two successive times, which is less than the half of wavelength (i.e., $\Delta d_{t}<\frac{\lambda }{2}$).

### 3.2. Clustering Laser Ranging Data

A 2D laser range finder is used to scan the environment and retrieve the distance and angle information of the moving object. We only save the laser ranging data when the reader detects the tag we want to track. Each scan point consists of a distance and angle measurement. In total, there are 450 ranging points in a scan cycle. In one scanning cycle, $\left(\rho_{n},\phi_{n}\right)$ represents the distance and angle of the *n*th laser beam [48,49] and can be described into Cartesian coordinate system:

(5) $$\left\{\begin{array}{c} x_{n}=\rho_{n}\cdot sin\phi_{n},\\ y_{n}=\rho_{n}\cdot cos\phi_{n}, \end{array}\right.$$

where $\left(x_{n},y_{n}\right)$ denotes the laser point $P_{n}$. The distance between the *i*th and *j*th laser point can be described as:

(6) $$d\left(P_{i},P_{j}\right)=\sqrt{{\left(x_{i}-x_{j}\right)}^{2}+{\left(y_{i}-y_{j}\right)}^{2}}.$$

**Figure 2:**
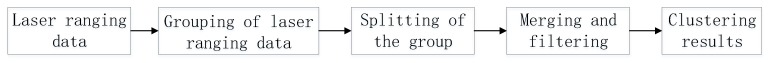
Overview of laser-based clustering.

**Figure 3:**
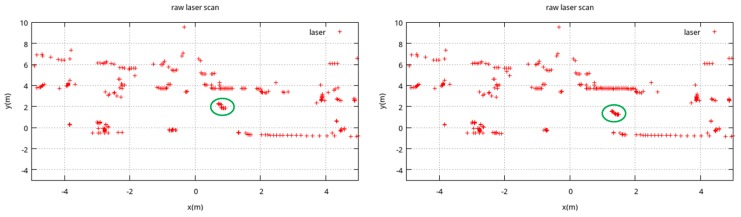
Laser ranging data at two timestamps.

For a better localization of the object, we segment the laser ranging data into different clusters. The process of clustering mainly includes: segmenting the raw data into groups (Section 3.2.1), splitting the group into clusters (Section 3.2.2), merging clusters (Section 3.2.3), and filtering out the ones with larger radii (Section 3.2.3). The final clustering results are considered as the obstacles [41,48,49]. The whole process of clustering is shown in Figure 2. Figure 3 is the raw laser ranging data at two nearby timestamps. As it can be seen from this figure, there are many obstacles in one environment. Therefore, we use the distance between two consecutive laser points for simple grouping, which will segment the laser ranging data into a group (the potential objects). We then split the group to improve the clustering accuracy.

#### 3.2.1. Grouping of Laser Ranging Data

**Figure 4:**
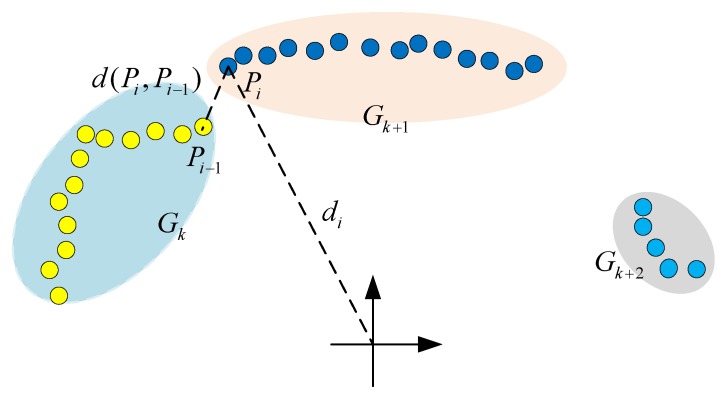
Illustration of laser-based grouping.

To perform clustering procedure, original laser ranging data is segmented into groups with the following rules: when the distance $d(P_{i},P_{i-1})$ between *i*th and the i−1th laser point satisfies Equation (7), we consider the two points as the same group $G_{k}$:

(7) $$d\left(P_{i},P_{i-1}\right)<d_{g}+d_{i}\cdot d_{p},$$

where $d_{g}$ is the threshold of grouping, $d_{i}$ is the distance between the *i*th laser point and robot, and $d_{p}$ is the distance parameter. When the continuous laser points (i.e., $P_{i}$ and $P_{i-1}$) in one scanning cycle do not satisfy Equation (7), a new group $G_{k+1}$ will be created based on point $P_{i}$. This step will lead to a grouping set $G_{k}\left(k\in \left\{1\cdots N_{g}\right\}\right)$, where $N_{g}$ is the number of groups. An example of the grouping result is shown in Figure 4.

#### 3.2.2. Splitting of the Group

**Figure 5:**
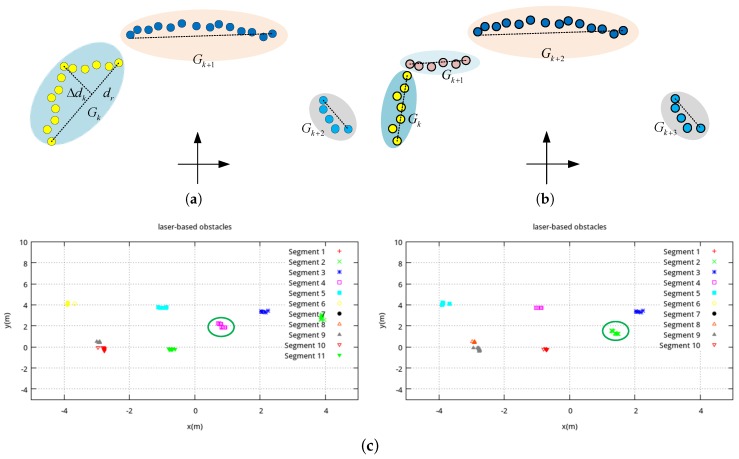
Comparison of the laser groups before and after splitting and a real example. (**a**) before splitting; (**b**) after splitting; (**c**) clustering results.

The grouping set $G_{k}\left(k=1\cdots N_{g}\right)$ is obtained by comparing the distance between the neighboring laser points, but two or more obstacles may exist in one group. Thus, we need to split the group in order to separate the obstacles. For this purpose, we use an iterated fitting algorithm. First, we find the two points in group $G_{k}$ with the longest distance and obtain the line connecting these two points. We then find the point with the longest perpendicular distance $\Delta d_{k}$ to the line. If $\Delta d_{k}$ satisfies Equation (8), then it continues the splitting process or otherwise quits the iteration. The splitting condition is defined as:

(8) $$\Delta d_{k}>d_{s}+d_{r}\cdot d_{p},$$

where $d_{s}$ is the threshold for splitting, and $d_{r}$ is length of the connecting line. If $G_{k}$ satisfies the splitting condition, we split $G_{k}$ into new groups as $G_{k}$ and $G_{k+1}$, as shown in Figure 5a,b. The iterated splitting process stops until there is no increase in the number of groups, We continue the split process until the number of clusters does not change. As a result, we obtain a number of clusters at time *t*:

(9) $$O_{t}=\left\{O_{t}^{\left(i\right)}\right\}={\left\{r_{t}^{\left(i\right)},o_{t}^{\left(i\right)}\right\}}_{i=1}^{N_{t}},$$

where $r_{t}^{\left(i\right)}=\frac{d_{r}}{2}$ is the radius of *i*th clustering, $o_{t}^{\left(i\right)}=\left(x_{t}^{\left(i\right)},y_{t}^{\left(i\right)}\right)$ is the center of *i*th cluster, and $N_{t}$ is the number of clusters at time *t*.

#### 3.2.3. Merging and Filtering

To avoid the overlapping between different clusters, we further merge the clusters with the following criteria:

(10) $$|r_{t}^{\left(i\right)}-r_{t}^{\left(j\right)}|\geq \sqrt{{\left(x_{t}^{\left(i\right)}-x_{t}^{\left(j\right)}\right)}^{2}+{\left(y_{t}^{\left(i\right)}-y_{t}^{\left(j\right)}\right)}^{2}}.$$

If Equation (10) is satisfied, the cluster $O_{t}^{\left(i\right)}$ and $O_{t}^{\left(j\right)}$ would be merged into one cluster. As our target is to localize common pedestrians, the radius of a cluster is confined by a threshold $r_{max}$. Finally, we use an example to demonstrate the process of laser clustering. Figure 3 is the raw laser scanning data at two consecutive timestamps, and Figure 5c is the clustering results.

### 3.3. Estimate the Distance-Based Velocity and Moving Direction of a Cluster

For each cluster $O_{t}^{\left(i\right)}$ at time *t*, we find the cluster $O_{t-1}^{\left(\hat{j}\right)}$ with a minimum distance at the previous time t−1. Therefore, ($O_{t}^{\left(i\right)},O_{t-1}^{\left(\hat{j}\right)}$) could be considered as the same object at two sequent times *t* and t−1. $\hat{j}$ is given by:

(11) $$\hat{j}=arg\min_{j}\sqrt{{\left(x_{t}^{\left(i\right)}-x_{t-1}^{\left(j\right)}\right)}^{2}+{\left(y_{t}^{\left(i\right)}-y_{t-1}^{\left(j\right)}\right)}^{2}},$$

where $1\leq j\leq N_{t-1}$. Therefore, the velocity and moving direction of the *i*th cluster can be computed as:

(12) $$v_{t}^{\left(i\right)}=\frac{\Delta d_{t}^{\left(i\right)}}{\Delta t}=\frac{\sqrt{{\left(x_{t}^{\left(i\right)}-x_{t-1}^{\left(\hat{j}\right)}\right)}^{2}+{\left(y_{t}^{\left(i\right)}-y_{t-1}^{\left(\hat{j}\right)}\right)}^{2}}}{\Delta t},$$

(13) $$\theta_{t}^{\left(i\right)}=atan\left(\frac{y_{t}^{\left(i\right)}-y_{t-1}^{\left(\hat{j}\right)}}{x_{t}^{\left(i\right)}-x_{t-1}^{\left(\hat{j}\right)}}\right).$$

To improve the accuracy of algorithm, we filter out the clusters having velocity higher than 1.0 m/s.

### 3.4. Similarity Computation Using Phase-Based and Distance-Based Velocity

Although the laser range finder can be used to obtain the distance and angle information, there is a singularity problem on the object identification. Due to the unique identification feature, RFID can precisely solve the singularity issue. We compare the similarity between the RFID phase-based velocity and the laser clustering-based velocity to evaluate whether both of them are from the same object or not. The similarity is computed as:

(14) $$sim\left(v_{t}^{\left(i\right)},v_{t}^{r}\right)=1-\frac{|v_{t}^{\left(i\right)}-v_{t}^{r}|}{|v_{t}^{\left(i\right)}+v_{t}^{r}|}.$$

In Equation (14), we can observe that, with a high similarity, there is more chance that the corresponding laser cluster is the object we want to localize. To improve the robustness of the system, we select the K clusters (i.e., $O_{t}^{m(1)},O_{t}^{m(2)},\cdots O_{t}^{m(K)}$) with best similarity values as the potential objects for updating weights of particles, which is described in Section 3.5.

### 3.5. Moving Object Localization with a Particle Filter Based on the K Best Clusters

In this section, we estimate the position of an object (i.e., posterior probability) by the probabilistic-based approach. The Bayesian inference is a probabilistic framework for estimating the probability density function over a state, given the past measurements reported by the sensor and the motions performed by the object. In our case, we use RFID and laser ranging measurement to estimate the state of the object, i.e., posterior probability density function of the state. According to Bayesian inference, we factorize the posterior probability $p(X_{t}|g_{1:t},r_{1:t},u_{1:t})$ into:

(15) $$p\left(X_{t}|g_{1:t},r_{1:t},u_{1:t}\right)=\eta_{t}\cdot p\left(X_{t}|X_{t-1},u_{t}\right)\cdot p\left(g_{t}|X_{t},r_{t}\right)\cdot p\left(X_{t-1}|g_{1:t-1},r_{1:t-1},u_{1:t-1}\right),$$

where $X_{t}$ is the position of the object, which we want to estimate at time *t*, $g_{t}$ is the measurement of the laser range finder at time *t*, $r_{t}$ is the measurement of RFID at time *t*, $u_{t}$ is the motion information of the object, and $\eta_{t}$ is a normalizer. $p(X_{t}|X_{t-1},u_{t})$ is the motion model, which is used to predict the object position at time *t* given the previous position $X_{t}$ and motion information $u_{t}$. $p(g_{t}|X_{t},r_{t})$ is the observation model, which describes the likelihood of receiving a measurement $g_{t}$ (i.e., laser-based clusters) given the RFID measurement (i.e., RFID phase-based velocity) and the current state $X_{t}$. Details of the motion model and observation model will be explained in Section 3.5.1 and Section 3.5.2, respectively. $p(X_{t-1}|g_{1:t-1},r_{1:t-1},u_{1:t-1})$ is the state at time t−1. To solve this nonlinear problem, we choose the particle filter as the implementation, due to its no-parametric features. Particle filter is a basic implementation of Bayesian framework, which uses a set of particles to approximate the posterior probability distribution. It has the advantages of high robustness and high accuracy to deal with nonlinear and non-Gaussian systems. In this paper, particle filter is used to fuse two kinds of information for positioning.

We represent the object location by a number of particles with different weights, i.e., $X_{t}={\left\{X_{t}^{\left[n\right]},\omega_{t}^{\left[n\right]}\right\}}_{n=1}^{N}$, where *N* is the number of particles, $X_{t}^{\left[n\right]}=\left\{x_{t}^{\left[n\right]},y_{t}^{\left[n\right]}\right\}$ denotes the 2D location of the particle, and $\omega_{t}^{\left[n\right]}$ is the associated weight [50]. The particle filter performs the following three steps based on the arrival of the sensor measurements: prediction, update, and resampling.

#### 3.5.1. Prediction

In this stage, we predict the state of a particle $X_{t}^{\left[n\right]}$ based on the previous state $X_{t-1}^{\left[n\right]}$ and the motion information of the object $u_{t}$. Here, to enhance the prediction performance, we use two predicting forms: random prediction and laser-based prediction. Random prediction is adopted when a matching cluster is not found, which is described as :

(16) $$\left\{\begin{array}{c} x_{t}^{\left[n\right]}=x_{t-1}^{\left[n\right]}+N\left(0,v_{t}^{r}\cdot \Delta t\cdot \sigma_{r}\right),\\ y_{t}^{\left[n\right]}=y_{t-1}^{\left[n\right]}+N\left(0,v_{t}^{r}\cdot \Delta t\cdot \sigma_{r}\right), \end{array}\right.$$

where $\sigma_{r}$ represents the random Gaussian noise. Random prediction can be considered as the complementary measurement when laser-based prediction is not available.

We use laser-based prediction when a valid matching cluster is found. In this way, we first find the nearest cluster *l* of particle *n*, then add Gaussian noise to its corresponding velocity $v_{t}^{\left(l\right)}$ and moving direction $\theta_{t}^{\left(l\right)}$. Finally, we execute the prediction according to $\tilde{v_{t}^{\left(l\right)}}$ and $\tilde{\theta_{t}^{\left(l\right)}}$:

(17) $$\tilde{\theta_{t}^{\left(l\right)}}=\theta_{t}^{\left(l\right)}+N\left(0,\sigma_{a}\right),$$

(18) $$\tilde{v_{t}^{\left(l\right)}}=v_{t}^{\left(l\right)}+N\left(0,\sigma_{v}\right),$$

(19) $$\left\{\begin{array}{c} x_{t}^{\left[n\right]}=x_{t-1}^{\left[n\right]}+\tilde{v_{t}^{\left(l\right)}}\cdot \Delta t\cdot cos\left(\tilde{\theta_{t}^{\left(l\right)}}\right),\\ y_{t}^{\left[n\right]}=y_{t-1}^{\left[n\right]}+\tilde{v_{t}^{\left(l\right)}}\cdot \Delta t\cdot sin\left(\tilde{\theta_{t}^{\left(l\right)}}\right), \end{array}\right.$$

where $\sigma_{a}$ and $\sigma_{v}$ represent the Gausian noises added to the velocity and the moving direction of the cluster *l*, respectively. In some cases where RFID and laser velocities do not match during positioning (when no matching cluster can be found), we use Equation (16) for prediction; otherwise, Equation (19) is used for prediction. Using two different prediction methods can improve the positioning accuracy.

#### 3.5.2. Update

The update stage is used to correct previous prediction by current measurement and update the weights of the particles. Based on the observation model $p(g_{t}|X_{t},r_{t})$, the weight $\omega_{t}^{\left[n\right]}$ of the particle $X_{t}^{\left[n\right]}$ is computed as:

(20) $$\omega_{t}^{\left[n\right]}=\eta_{t}\cdot \omega_{t-1}^{\left[n\right]}\cdot p\left(g_{t}|X_{t}^{\left[n\right]},r_{t}\right).$$

Combined with the weighted *K* most similar clusters, the observation model $p(g_{t}|X_{t},r_{t})$ is approximated as:

(21) $$p\left(g_{t}|X_{t},r_{t}\right)=\sum_{i=1}^{K}sim\left(v_{t}^{m(i)},v_{t}^{r}\right)\cdot \exp\left(-\frac{d^{2}\left(X_{t}^{\left[n\right]},O_{t}^{m(i)}\right)}{2}\right),$$

where

(22) $$d^{2}\left(X_{t}^{\left[n\right]},O_{t}^{m(i)}\right)=\frac{{\left(x_{t}^{\left[n\right]}-x_{t}^{m(i)}\right)}^{2}}{\sigma_{d}}+\frac{{\left(y_{t}^{\left[n\right]}-y_{t}^{m(i)}\right)}^{2}}{\sigma_{d}},$$

where $\sigma_{d}$ is a bandwidth parameter added to the distance to the cluster. The impact of $\sigma_{d}$ on the positioning accuracy is analyzed in the experimental section (Section 4.2.6).

#### 3.5.3. Resampling

We resample the particles to ensure the efficiency of particle filter. Resampling generates a set of new particles depending on the weights of the particles. The weights of the particles change after the particles are updated, in which the particles that are far away from the target have the smaller weight and the ones closer the target have the larger weight. After many iterations, some particle weights become very small, which is the phenomenon of particle degeneration. In order to effectively reduce the phenomenon of particle degeneration, we use resampling to remove the less weighted particles and duplicate the particles with large weights.

## 4. Experimental Results

### 4.1. Experimental Setups

**Figure 6:**
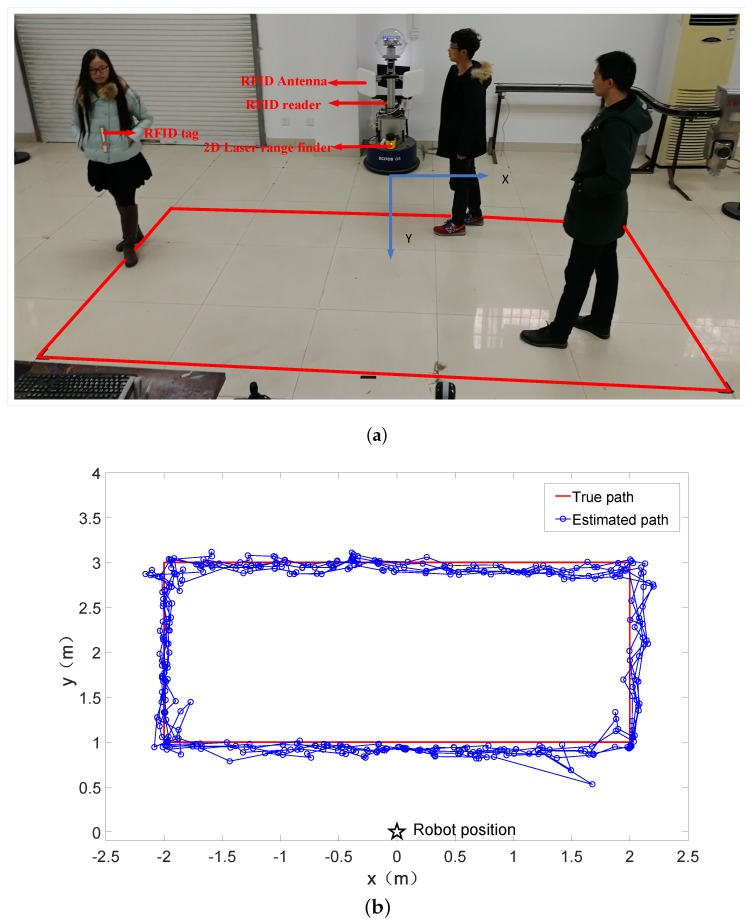
Setup of the experiment and positioning result. (**a**) Setup of the experiment; (**b**) ground truth and estimated track by a combination of two prediction forms.

The experiments to check the feasibility of our approach were performed on a service robot SCITOS G5 from Metralabs GmbH, Ilmenau, Germany), as shown in Figure 6. The robot is equipped with a 2D laser range finder (SICK S300), a UHF RFID reader (Speedway Revolution R420 from Impinj, Inc., Seattle, WA, USA) with a sampling frequency of 2 Hz, and two circularly polarized antennas (RFMAX SS8688P from Laird Technologies, London, UK) at two sides of the robot with angles of $45^{\circ }$. The RFID reader offers a reading range up to 7 m. During the experiment, we use Dense Reader Mode 8 (DRM8) and a channel of 920.625 MHz for the RFID reader. The RFID antenna is placed at the same height as the tag (1.2 m above the ground). The measuring range for the laser range finder is up to 29 m, within the angle of $270^{\circ }$ and resolution of $0.5{}^{\circ }$. In addition, the laser range finder works at a frequency of 20 HZ. During the experiments, we affixed a tag (Alien Squiggle RFID Wet Inlay from Alien Technology, San Jose, CA, USA) on a moving object.

We set the testing space for positioning by a rectangular area of 4 m × 2 m, and the robot was placed one meter perpendicular to the long side of the rectangle, as shown in Figure 6a. A person carried the tag and walked along the edge of rectangular area for 5 rounds at the velocity of approx. 0.4 m/s to test the positioning accuracy.

**Figure 7:**
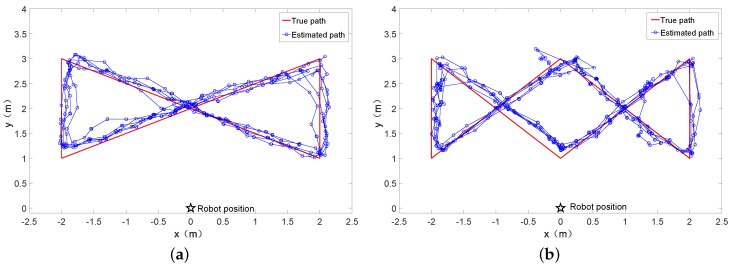
Complex paths. (**a**) 8-shaped path; (**b**) W-shaped path.complex paths

The comparison between the estimated path and the true path is depicted in Figure 6b. In addition, we walked the more complex paths in the test area to test the usability of our approach. The results are shown in Figure 7a,b. In order to facilitate comparison, we use the rectangular path as the adjustment of the experimental parameters. From these figures, we can see that the estimated path is basically consistent to the true path, which demonstrates the feasibility of our approach. The final result shows that mean positioning error is 0.25 m. Average positioning error means the RMSE (rooted mean square error). The error is defined as the Euclidean distance between the ground truth and the estimated position.

### 4.2. Impact of Different Parameters on the Positioning Accuracy

Next, we carried out six different experiments for evaluating the positioning accuracy under the impact of different factors in each experiment, including antenna configurations, predicting forms, factors in clustering (i.e., $d_{g}$, $d_{p}$, $d_{s}$, and $r_{max}$), the number of particles *N*, noise settings (i.e., $\sigma_{d}$ and $\sigma_{a}$ ), *K* value, and factor of $\sigma_{d}$.

#### 4.2.1. Impact of Different Antenna Configurations

As the detecting range of RFID is directly confined by antennas on robot, in the first series of experiments, we analyzed the positioning accuracy under different antenna combinations. We fixed $d_{s}=0.1$, $r_{max}=1.0$, $d_{p}=0.01$, $d_{g}=0.2$, N=100, $\sigma_{a}=0.1$, $\sigma_{v}=1.0$, K=4, and $\sigma_{d}=0.1$. The result is shown in Table 2. As it can be seen from this table, the positioning accuracy is impacted by the number of antennas used. The accuracy is low while using only one antenna due to its incompleteness on coverage, while we obtain a better positioning accuracy with two antennas as the error decreased from 1.24 m to 0.258 m (i.e., 79% improvement). Therefore, this paper uses two antennas to locate the moving object.

#### 4.2.2. Impact of Different Prediction Forms

**Figure 8:**
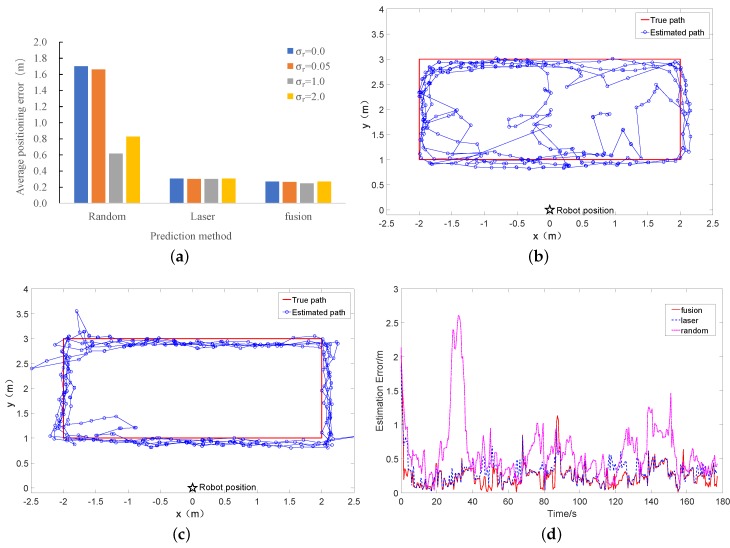
Comparison of the performance in three different prediction forms. (**a**) mean positioning accuracy under different prediction forms; (**b**) ground truth and estimated track of random prediction; (**c**) ground truth and estimated track of laser prediction; (**d**) estimation error at different timestamps.Different prediction method.

In the next series of experiments, we tested the impact of positioning accuracy under three different predicting forms: the random predicting, laser predicting, and the combination of random predicting and laser predicting. The experimental settings were the same as described previously. The results are shown in Figure 6b and Figure 8. As it can be seen from this figure, the choice of $\sigma_{r}$ has a significant impact on the positioning accuracy for the random prediction. $\sigma_{r}=1.0$ leads to the best positioning accuracy, while a too small or too large $\sigma_{r}$ obviously leads to a worse positioning result (1.7 m for $\sigma_{r}=0.0$ and 0.829 m for $\sigma_{r}=2.0$, as compared to 0.618 m for $\sigma_{r}=1.0$). The reason is that, with a small $\sigma_{r}$, all particles will converge to a single point after the integration of several RFID and laser measurements, while, with a large $\sigma_{r}$, the particle filter is not able to converge, due to the large noise added to the particles.

In addition, as can be seen from Figure 6b and Figure 8, the combining way of prediction was better than the random prediction or the prediction based on laser information, with the locating error diminished from 0.618 m to 0.248 m (59% improvement) and 0.302 m to 0.248 m (18% improvement), respectively. Figure 6b and Figure 8b–c show the track using the three different predicting forms. Figure 8d shows the real-time error of three different prediction forms.

#### 4.2.3. Impact of Different Parameters of Laser Clustering

**Figure 9:**
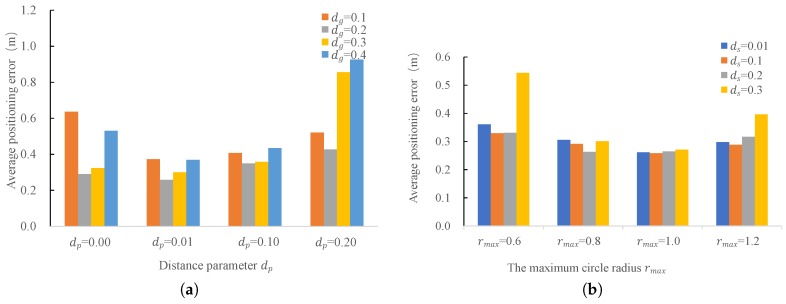
Positioning accuracy under the impact of different parameters of laser clustering. (**a**) Impact of different grouping thresholds $d_{g}$ and distance parameters $d_{p}$; (**b**) Impact of different maximum cluster radius $r_{max}$ and splitting threshold $d_{s}$.

In the next series of experiments, we compared the positioning accuracy under the impact of different settings of the laser clustering, i.e., grouping threshold $d_{g}$, distance parameter $d_{p}$, splitting threshold $d_{s}$, and the radius threshold $r_{max}$. The results are shown in Figure 9. As can be seen from Figure 9a, grouping threshold $d_{g}$ and the distance parameter $d_{p}$ have high impact on the positioning accuracy. The accuracy decreases when $d_{g}$ and $d_{p}$ are too small or too large. A choice of $d_{g}=0.2$ and $d_{p}=0.01$ gives the best positioning accuracy. As can be seen from Figure 9b, the radius threshold $r_{max}$ also has some impact on the positioning accuracy. A too small $r_{max}$ will retain many useless clusters and introduces more error to the positioning, while a too large $r_{max}$ will filter out the true clusters and gives a poor positioning accuracy as well. As can also be seen from Figure 9b, the positioning accuracy decreases with a too small or too large splitting threshold $d_{s}$. When the splitting threshold $d_{s}$ is too small (for example $d_{s}=0.01$), one obstacle is segmented into several groups and leads to an increase of positioning error, while the accuracy gets worse with a too large $d_{s}$, as several obstacles are segmented into one group. To keep all these analysis in mind, we choose the parameters as: $d_{p}=0.01$, $d_{g}=0.2$, $d_{s}=0.1$, and $r_{max}=1.0$.

#### 4.2.4. Impact of Different Number of Particles *N*

As the number of particles is directly related to the processing time and positioning accuracy, we carried out experiments to analyse the impact of the number of particles. We used a CPU with Core i3-2330M, 2.2GHz and 6 GB RAM for the processing. Other parameters are set to be the same as previously and the system is checked for different numbers of particles. The results are listed in Table 3. From this table, we can observe that a small number of particles (i.e., N<100) leads to a poor positioning accuracy. The positioning accuracy increases (but not so obviously) with the increase of the number of particles, but the processing time increases significantly at the same time. Therefore, we choose a particle number of N=100 to achieve the better positioning accuracy and algorithm efficiency.

#### 4.2.5. Comparison of Different Velocity Noise $\sigma_{v}$ and Moving Direction Noise $\sigma_{a}$

**Figure 10:**
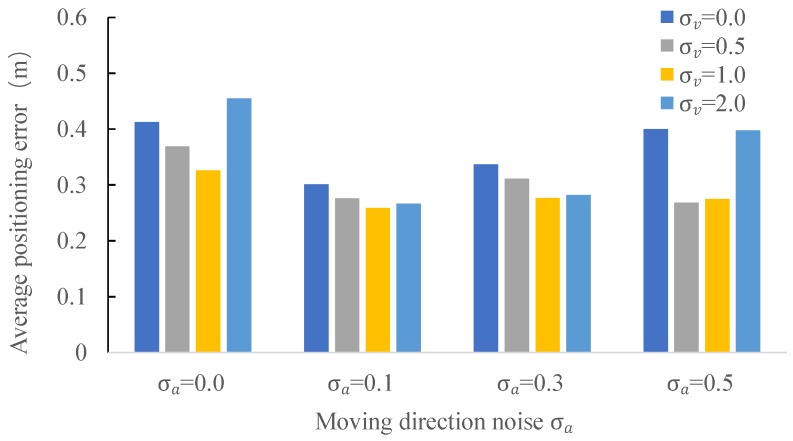
Positioning accuracy under the impact of different velocity noise $\sigma_{v}$ and moving direction noise $\sigma_{a}$.

In the next series of experiments, we evaluated the impact of the Gaussian noise $\sigma_{v}$ and $\sigma_{a}$ added to the velocity and moving direction of laser clusters. We use the same parameter settings as previously except the ones mentioned below and show the results in Figure 10. As can be seen from this figure, a too large or too small $\sigma_{a}$ will result in a decrease of the positioning accuracy. We get the best positioning accuracy (i.e., 0.259 m) with $\sigma_{a}=0.1$ for a setting of $\sigma_{v}=1.0$, which is an improvement of 21.5% and 7.5%, as compared to $\sigma_{a}=0.0$ (0.33 m) and $\sigma_{a}=0.5$ (0.28 m), respectively. The same findings apply to the parameter of $\sigma_{v}$. Based on the results in Figure 10, we chose $\sigma_{a}=0.1$ and $\sigma_{v}=1.0$ to achieve the best accuracy of the system.

#### 4.2.6. Impact of Different *K* and the Bandwidth Parameter $\sigma_{d}$

**Figure 11:**
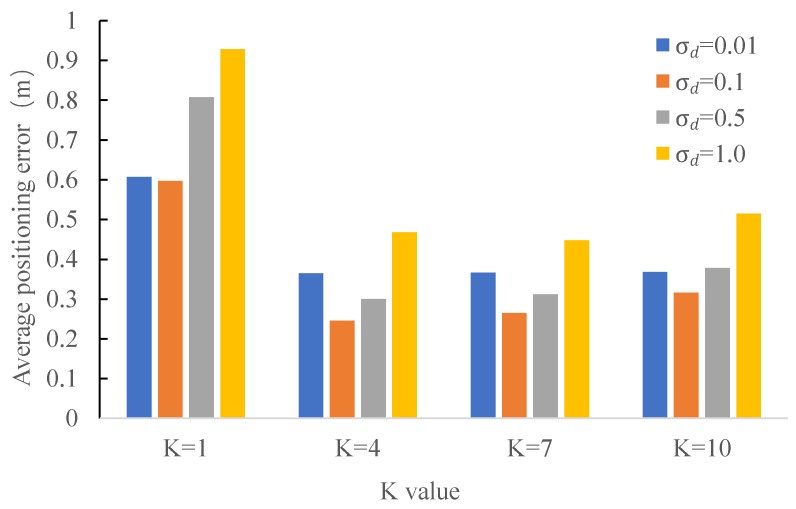
Mean positioning error under the impact of different *K* and bandwidth $\sigma_{d}$.

In the last series of experiments, we tested the positioning accuracy under the impact of different settings of *K* and the bandwidth parameter $\sigma_{d}$. The results are shown in Figure 11. As it can be seen from this figure, K=4 gives the best result. A too large or too small *K* leads to a worse positioning result. Moreover, we also find a suitable value of $\sigma_{d}$ is at around 0.1. The positioning accuracy gets worse when $\sigma_{d}$ is too large (e.g., 1.0) or too small (e.g., 0.01). With the best settings of *K* and $\sigma_{d}$ (i.e., K=4 and $\sigma_{d}=0.1$), we achieve a positioning accuracy of 0.25 m.

## 5. Conclusions

In this paper, we proposed a new approach for moving object localization based on RFID phase and laser clustering. We first computed the RFID phase-based velocity by the phase difference and laser-based velocity from laser clustering. Then, a particle filter is used for the sensor fusion based on the best K matching clusters. We conducted extensive experiments to validate the feasibility of the proposed approach and our results show that a mean positioning accuracy up to 0.25 m can be achieved. In future, we would like to increase the positioning accuracy by incorporating the signal strength information and extend our work to include multiple moving objects.

## Figures and Tables

**Table 1:** Mathematical symbols and their meanings.

Mathematical Symbol	Meaning
$\varphi_{t}$	Phase of RFID signal at time *t*
$v_{t}^{r}$	Phase-based velocity of RFID tag at time *t*
$d_{g}$	Grouping threshold in laser-based clustering
$d_{p}$	Distance parameter in laser-based clustering
$d_{s}$	Splitting threshold in laser-based clustering
$r_{max}$	The maximum cluster radius
$v_{t}^{\left(i\right)}$	The velocity of cluster *i* at time *t*
$\theta_{t}^{\left(i\right)}$	The moving direction of cluster *i* at time *t*
*K*	The number of the best matching clusters
*N*	Number of particles
$X_{t}$	The object position at time *t*
$\left(x_{t}^{\left[n\right]},y_{t}^{\left[n\right]}\right)$	Location of particle *n* at time *t*
$\omega_{t}^{\left[n\right]}$	The weight of particle *n* at time t
$\sigma_{r}$	Gaussian noise in random prediction
$\sigma_{a}$	Gaussian noise added to the moving direction in laser prediction
$\sigma_{v}$	Gaussian noise added to the velocity in laser prediction
$\sigma_{d}$	The bandwidth parameter used to control the weight update of the particle filter
$\tilde{\theta_{t}^{\left(l\right)}}$	Moving direction after adding Gaussian noise $\sigma_{a}$ to the cluster *l* at time *t*
$\tilde{v_{t}^{\left(l\right)}}$	Velocity after adding Gaussian noise $\sigma_{v}$ to cluster *l* at time *t*

**Table 2:** Comparison of positioning accuracy in meters under different antenna combinations.

Antenna Combination	Only Right Antenna	Only Left Antenna	Right and Left Antennas
Positioning accuracy (m)	1.24	1.41	0.258

**Table 3:** Mean positioning accuracy and running time of the algorithm under the impact of different number of particles *N*.

Number of Particles *N*	Accuracy (m)	Running Time (ms)
5	0.457	4.187
10	0.295	4.535
50	0.279	4.655
100	0.256	4.858
500	0.254	6.654
1000	0.258	8.594

## Abbreviations

The following abbreviations are used in this manuscript:

UHF	Ultra High Frequency
LBS	Location-Based Services
RFID	Radio Frequency Identification
Wi-Fi	Wireless Fidelity
RSS	Received Signal Strength
TOA	Time of Arrival
AOA	Angel of Arrival
WSN	Wireless Sensor Network
EKF	Extended Kalman Filter
VPH	Vector Polar Histogram
RMSE	Rooted Mean Square Error
